# Linking Late Endosomal Cholesterol with Cancer Progression and Anticancer Drug Resistance

**DOI:** 10.3390/ijms23137206

**Published:** 2022-06-29

**Authors:** Mai K. L. Nguyen, Jaimy Jose, Mohamed Wahba, Marc Bernaus-Esqué, Andrew J. Hoy, Carlos Enrich, Carles Rentero, Thomas Grewal

**Affiliations:** 1 School of Pharmacy, Faculty of Medicine and Health, University of Sydney, Sydney, NSW 2006, Australia; mngu0111@uni.sydney.edu.au (M.K.L.N.); jjos6151@uni.sydney.edu.au (J.J.); mwah4790@uni.sydney.edu.au (M.W.); 2 Departament de Biomedicina, Unitat de Biologia Cel·lular, Facultat de Medicina i Ciències de la Salut, Universitat de Barcelona, 08036 Barcelona, Spain; marcbernaus@ub.edu (M.B.-E.); enrich@ub.edu (C.E.); carles.rentero@ub.edu (C.R.); 3 Centre de Recerca Biomèdica CELLEX, Institut d’Investigacions Biomèdiques August Pi i Sunyer (IDIBAPS), 08036 Barcelona, Spain; 4 School of Medical Sciences, Charles Perkins Centre, Faculty of Medicine and Health, University of Sydney, Sydney, NSW 2006, Australia; andrew.hoy@sydney.edu.au

**Keywords:** cancer, cholesterol transporters, late endosomes/lysosomes, LDL-cholesterol, NPC1, Rab7, StARD3, Annexin A6

## Abstract

Cancer cells undergo drastic metabolic adaptions to cover increased bioenergetic needs, contributing to resistance to therapies. This includes a higher demand for cholesterol, which often coincides with elevated cholesterol uptake from low-density lipoproteins (LDL) and overexpression of the LDL receptor in many cancers. This implies the need for cancer cells to accommodate an increased delivery of LDL along the endocytic pathway to late endosomes/lysosomes (LE/Lys), providing a rapid and effective distribution of LDL-derived cholesterol from LE/Lys to other organelles for cholesterol to foster cancer growth and spread. LDL-cholesterol exported from LE/Lys is facilitated by Niemann–Pick Type C1/2 (NPC1/2) proteins, members of the steroidogenic acute regulatory-related lipid transfer domain (StARD) and oxysterol-binding protein (OSBP) families. In addition, lysosomal membrane proteins, small Rab GTPases as well as scaffolding proteins, including annexin A6 (AnxA6), contribute to regulating cholesterol egress from LE/Lys. Here, we summarize current knowledge that links upregulated activity and expression of cholesterol transporters and related proteins in LE/Lys with cancer growth, progression and treatment outcomes. Several mechanisms on how cellular distribution of LDL-derived cholesterol from LE/Lys influences cancer cell behavior are reviewed, some of those providing opportunities for treatment strategies to reduce cancer progression and anticancer drug resistance.

## 1. Introduction

Lipids represent a diverse family of biomolecules responsible for a plethora of cellular functions, including the provision of building blocks for membrane integrity, energy storage, and serving as precursors for hormones, vitamins and signalling molecules. Hence, cellular lipid metabolism is highly coordinated, and controlled by signalling networks, transcription factors and feedback mechanisms, ensuring a proper response to cellular nutrient status that is in sync with overall body physiology. However, in chronic diseases such as cancer, cells undergo metabolic adaptations that reflect or accommodate pathophysiological changes [1,2]. This comprises alterations in lipid metabolism, including cholesterol homeostasis, and understanding the lipid-related molecular changes associated with disease progression can contribute to improving therapeutic approaches [3,4].

The large and highly varied group of lipids consists of fatty acids, triglycerides, phospholipids, sphingo- and glycolipids, as well as sterols [5]. The latter entails cholesterol, a 27-carbon polycyclic lipid molecule first isolated from human gallstones, with prominent roles in cellular homeostasis that have physiological significance in health and disease [3,4]. In particular, cholesterol is an indispensable constituent of cellular membranes, responsible for maintaining membrane integrity, compartmentalization and proper functioning of organelles, cell surface receptors and protein complex assembly driving signal transduction, while also serving as a precursor for bile acids, steroid hormones and vitamins [3,4,6].

**Figure 1:**
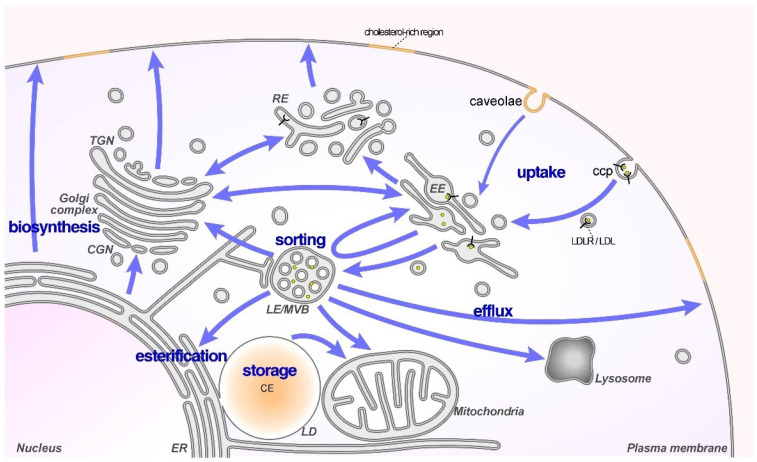
Late endosomes/multivesicular bodies (LE/MVB) are the hub for the cellular distribution of cholesterol. LDLR-mediated endocytosis delivers LDL-derived cholesterol to LE/MVB from where cholesterol is distributed via Niemann–Pick type C1/2 (NPC1/2) proteins and other transporters to the ER, mitochondria, Golgi and plasma membrane. The cellular transport routes of cholesterol are indicated. Abbreviations: CE, cholesteryl ester; ccp, clathrin-coated pit; EE/RE, early/recycling endosomes; ER, endoplasmic reticulum; LD, lipid droplet; LDL, low-density lipoprotein; LDLR, LDL receptor; MVB, multivesicular bodies; TGN, trans-Golgi network.

Although most mammalian cells are equipped with the machinery to synthesize cholesterol, feedback control mechanisms limit utilization of this energy-consuming process and most cells acquire cholesterol via receptor-mediated endocytosis of low-density lipoproteins (LDL) from plasma. Following the internalization of the LDL/LDL receptor (LDLR) complex, LDL is delivered to early endosomes and then targeted to late endosomes (LE)/lysosomes (Lys) [7,8]. The increasingly acidic environment in the LE/Lys lumen leads to the dissociation of LDL from its receptor and LDL-derived cholesteryl esters are then hydrolyzed to free cholesterol by lysosomal lipases. Cholesterol transporters in LE/Lys, in particular, Niemann–Pick type C1/2 (NPC1/2) proteins, then facilitate transport of late endosomal/lysosomal cholesterol (LE/Lys-Chol) to other cellular sites [7,8]. Besides NPC1/2, the steroidogenic acute regulatory (StAR)-related lipid transfer domain containing 3 (StARD3), StARD3 N-terminal like (StARD3NL), members of the oxysterol-binding protein (OSBP) family, such as OSBP-related proteins (ORP1L, ORP2), and lysosomal membrane proteins (lysosomal associated membrane protein 2 (LAMP2), lysosomal integral membrane protein 2 (LIMP2)) [9,10,11,12], contribute to LE/Lys-Chol transport. Together with several small Rab GTPases (Rab7, 8, 9 and 11) and scaffolding proteins (annexins), this ensures the delivery of cholesterol to the plasma membrane, the endoplasmic reticulum (ER), Golgi and mitochondria, required for the proper functioning of many cellular processes in these compartments [9,10,11,12] (Figure 1). As high amounts of free cholesterol are toxic to cells, LE/Lys-Chol can be transferred to the ER for esterification by acetyl-CoA cholesteryl acyltransferase 1 (ACAT1) and subsequent storage in lipid droplets. Alternatively, excess LE/Lys-Chol can be delivered to the cell surface and effluxed [9,10,11,12].

In this review, we will provide an overview of the existing knowledge that implicates the export of LDL-derived cholesterol from LE/Lys mediated by NPC1/2, other cholesterol transporters and related proteins in LE/Lys with cancer cell growth, metastatic behavior and anticancer drug efficacy. Transport routes of LDL-derived cholesterol to other cellular sites, and potential underlying mechanisms at these destinations enabling cholesterol from endocytosed LDL to modulate cancer cell activities are provided and discussed, Furthermore, several examples for treatment strategies targeting cholesterol export from LE/Lys to reduce cancer progression and anticancer drug resistance are listed.

## 2. LDL-Cholesterol: A Risk Factor for Cancer

Like other chronic diseases, the reprogramming of lipid metabolism is common in human cancers. These metabolic adaptations include mechanisms that cover the increased demand for cholesterol to support cancer growth and progression, and contribute to resistance to antitumoral therapies [1,2,13,14].

For cancer cells to accommodate the increased need for cholesterol, de novo cholesterol synthesis is often upregulated. Alternatively, elevated serum cholesterol levels can improve cholesterol availability for tumor growth and progression, contributing to cholesterol accumulation observed in many cancers [15,16]. For instance, increased plasma LDL levels were associated with an elevated lung, pancreatic and breast cancer risk [17]. Likewise, elevated serum cholesterol correlated with a higher risk of prostate cancer development and progression and hypercholesterolemia was associated with a shorter time to the development of castrate-resistant prostate cancer [13,18]. In addition, diet or obesity-induced development of cholesterol-rich environments are risk factors for tumor initiation and progression, with the administration of a western diet showing accelerated mammary tumor onset, increased tumor incidence, multiplicity and burden in mouse models [19].

### Increased LDL-Cholesterol Uptake Supports Cancer Growth and Progression

Elevated LDL uptake is common in cancer cells and provides a source for cholesterol to support many biological processes that are upregulated in oncogenic settings. It would go beyond the scope of this review to list all cholesterol-related functions that contribute to cancer cell growth and progression, and we refer the reader to excellent review articles [3,4,15,16]. In brief, cholesterol serves as a building block for membranes in proliferating cells. Along secretory pathways, and at the cell surface, cholesterol supports the formation of specialized membrane microdomains (e.g., lipid rafts, focal adhesions) responsible for oncogenic signalling, adhesion, migration and invasion. Furthermore, the physical interaction of cholesterol with cell surface receptors, such as the smoothened receptor [20], PDZ scaffolding proteins [21], soluble NSF attachment protein receptor (SNARE) proteins [10] or protein complexes that regulate mammalian target of rapamycin complex 1 (mTORC1) kinase [22] (see also Section 3.1 and Section 3.2) can also drive proliferation, invasion and metastasis. In addition, increased cholesterol levels in mitochondria are anti-apoptotic, contribute to chemoresistance, and support the ability of mitochondria to generate energy and synthesize steroid hormones to foster growth and progression in several cancers [23].

Effectively increasing the capacity to endocytose LDL, LDLR overexpression has been documented in pancreatic ductal adenocarcinoma (PDAC) [24,25], breast cancer [26,27], hepatocellular carcinoma (HCC) [28], lymphoma [29], lung adenocarcinoma [30,31], colorectal carcinoma [32,33], nasopharyngeal carcinoma [34], glioblastoma [35], leukemia [36] and renal cell carcinoma [37] (Table 1). Of note, in some cancer patients, this coincides with low LDL-cholesterol plasma levels [38], and even hypocholesterolemia in acute myeloid leukemia (AML) [39,40], prostate, lung, bowel, head and neck cancers [41,42,43], implicating enhanced LDL-cholesterol uptake by tumor cells causing reduced plasma cholesterol levels. Likewise, LDL clearance was greatly increased during prostate and lung cancer progression [44,45], suggesting LDL-cholesterol supports metastatic behavior. Despite these findings, and possibly due to an increased reliance on upregulated cholesterol biosynthesis, others have correlated low LDLR levels with poor prognosis and clinical outcomes in HCC, prostate and cervical cancer [46,47,48].

In support of the conclusions drawn from the majority of the abovementioned patient data, increased LDL uptake and elevated LDLR expression promoted growth in breast [52], prostate [54] and colorectal cancer models [32]. Vice versa, the silencing of LDLR expression or blocking of LDLR activity inhibited proliferation in pancreatic [25], prostate [53,55], nasopharyngeal [34], colon [32] and breast cancer models [54]. In prostate and pancreatic cancer cell lines and tissues, increased cholesteryl ester storage upon LDL exposure served as a reservoir for fatty acid and cholesterol to assist in tumor growth and progression [13,60,61] and was associated with elevated ACAT1 and LDLR levels [26]. Along these lines, ACAT1 inhibition reduced LDL-inducible proliferation, migratory behavior and restored feedback downregulation of LDLR [13,61].

Hence, LDLR expression might even serve as a prognostic marker to determine clinical outcomes. In fact, LDLR levels are inversely correlated with overall and recurrence-free survival in AML [55], breast cancer [38] and PDAC [53,61]. Elevated LDLR levels were also associated with an increased risk of PDAC recurrence [15,16] and a higher incidence of relapse in AML [55]. Strikingly, in mouse models of hyperlipidemia, elevated LDLR expression accelerated breast cancer growth and was associated with reduced efficiency of systemic therapies [38]. LDLR expression was also higher in epithelial ovarian carcinomas and leukemic cells that were resistant to platinum-based chemotherapy. In contrast, LDLR knockdown sensitized epithelial ovarian carcinomas to cisplatin [60] and potentiated gemcitabine-induced regression in pancreatic cancer cells [16] (Table 1).

## 3. Cholesterol Transporters in LE/Lys Contribute to Cancer Cell Behavior

**Figure 2:**
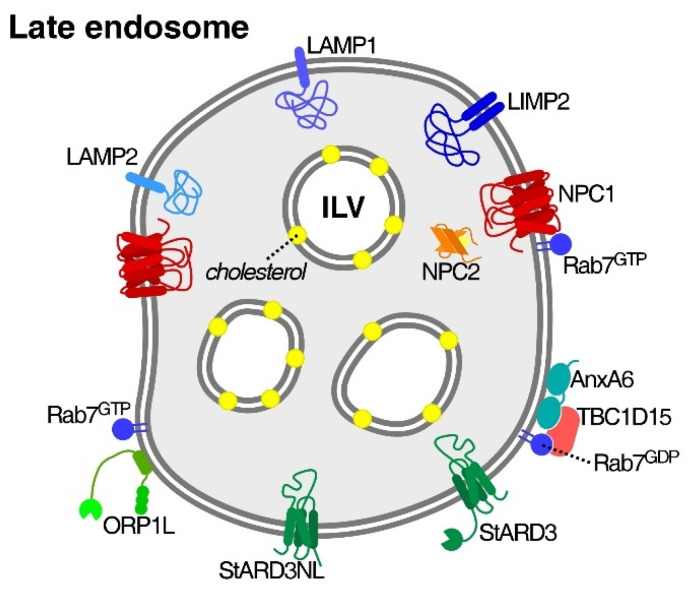
Cholesterol transporters and other proteins contributing to cholesterol export from late endosomes. Abbreviations: ILV, intraluminal vesicles; LAMP1/2, lysosome-associated membrane proteins 1/2; LIMP2, lysosomal integral membrane protein 2; NPC1/2, Niemann–Pick Type C1/2; AnxA6, annexin A6; TBC1D15, Tre-2/Bub2/Cdc16 domain family member 15; ORP1L, OSBP-related protein 1L; StARD3, Steroidogenic acute regulatory (StAR)-related lipid transfer domain containing 3; StARD3NL, StARD3 N-terminal-like protein.

Increased LDL endocytosis supporting cancer growth and progression (see Section 1 and Section 2) implies that cancer cells adapt and install mechanisms that can take advantage of an increased delivery of lipid cargo to LE/Lys, the cellular hub responsible for the distribution of LDL-derived cholesterol. However, the contribution to oncogenesis and tumor progression of molecular players downstream of the LDL/LDLR axis and accountable for cholesterol distribution from LE/Lys to other organelles is far less defined (Figure 2). In the following, we summarize key findings on the contribution of cholesterol transporters and regulatory proteins in LE/Lys to cancer growth, metastasis and drug resistance.

### 3.1. Niemann–Pick Type C1/2 (NPC1/2) Proteins

NPC1/2 proteins are critical for cholesterol export from LE/Lys, as loss-of-function mutations in the NPC1 or NPC2 gene cause NPC disease, a fatal neurodegenerative lysosomal storage disorder. In the absence of NPC1/2, cholesterol accumulates within the LE/Lys lumen, leading to a cellular cholesterol imbalance that derails membrane trafficking and communication between all other organelles, ultimately causing cellular dysfunction and death [9,10,11]. Mechanistically, NPC2 in the LE/Lys lumen binds and delivers cholesterol to NPC1 in the LE/Lys-limiting membrane [62,63]. NPC1, likely in cooperation with other proteins, then delivers cholesterol to the plasma membrane and directly or via the plasma membrane to other organelles, including the ER, Golgi, mitochondria and recycling endosomes [8,9,10,11,12].

Several observations have associated NPC1 expression patterns with cancer. Elevated NPC1 levels have been correlated with decreased overall survival in glioma [64] and a high risk of esophageal cancer [65]. NPC1 was also upregulated in metastatic estrogen receptor (ER)-negative breast cancer cells [66], indicating efficient LE/Lys-Chol distribution to support cancer progression. In contrast, NPC1 knockdown, loss-of-function or overexpression of a dominant-negative NPC1 mutant that cannot bind cholesterol inhibited the proliferation, spreading and migration of several common cancer cell lines [67,68,69,70,71]. Elevated NPC1 levels in imatinib- and daunorubicin-resistant leukemic cells were considered to undermine anticancer therapies, as NPC1 possibly supported efflux of these anticancer drugs [72,73]. In addition, in esophageal adenocarcinoma, two biopsies contained a fusion transcript of NPC1 with a mitotic kinase, maternal embryonic leucine zipper kinase [74]. As the accumulation of cholesterol and other lipids can lead to liver injury during chronic inflammation, NPC patients may also be more susceptible to the risk of fibrosis, cirrhosis and, ultimately, HCC development [75] (Table 2).

Excitingly, the repurposing of approved drugs has revealed the potential of targeting NPC1, disrupting cholesterol egress from LE/Lys, to prevent tumor growth and metastasis. Itraconazole, an azole antifungal agent, binds and inhibits NPC1, causing LE/Lys-Chol accumulation [77,80,85,86]. In several cancer models, itraconazole downregulated mTORC1 signalling, the key mediator of cancer cell metabolism, and diminished angiogenesis and tumor growth [78,85,86,87]. Itraconazole potentiated the antitumor effects of cisplatin [80], and entered Phase I and II clinical trials for non-small cell lung cancer [78], basal cell carcinoma [77], metastatic prostate [80] and pancreatic cancer [79]. In addition, the naturally occurring alkaloid cepharanthine used to treat acute and chronic diseases and the antihistamine astemizole interfered with NPC1-dependent LE/Lys-Chol egress, which correlated with downregulated mTORC1 activation, proliferation and migration of endothelial cells, and improved the efficacy of anticancer agents and alleviation of chemotherapy-induced adverse effects [81,82,88]. In addition, leelamine, a lipophilic diterpene amine and lysosomotropic compound that can interact with NPC1, inhibited LE/Lys-Chol export. This correlated with effective inhibition of signalling cascades that promote cancer cell survival and metastasis [83,84] (Table 2).

### 3.2. Cholesterol-Sensitive Mechanisms in LE/Lys That Influence Cancer Cell Activities

A number of mechanisms may affect cancer cell comportment in response to NPC1-mediated LE/Lys-Chol transport. At least in cell-based models, one prominent molecular target of NPC1 within close proximity to the LE/Lys limiting membrane is mTORC1, the master growth regulator commonly upregulated in cancer cell metabolism [22,89,90,91], promoting anabolic processes that support growth and proliferation. In response to intracellular nutrients, cytosolic mTORC1 kinase is recruited to LE/Lys and activated by growth factor-induced phosphatidylinositol 3-kinase (PI3K)/protein kinase B (AKT) signalling. Recently, cholesterol entering LE/Lys, either through LDL endocytosis or transfer across ER-lysosome contacts, was identified as a nutrient that can promote mTORC1 activation. This is achieved by LE/Lys-Chol interacting with mTORC1 regulators, heterodimeric Rag GTPases, their membrane anchor Ragulator and the lysosomal amino acid permease SLC38A9 [22,89]. In some cell models, NPC1 appeared to antagonize mTORC1 activation, as cholesterol accumulation upon NPC1 inhibition led to mTORC1 hyperactivation [89]. In addition, the crosstalk between NPC1 and mTORC1 is further complicated through NPC1 influencing Akt signalling, which is upstream of the mTORC1 complex and frequently activated in human cancers [67]. Hence, future studies still need to resolve if NPC1-dependent cholesterol egress across the LE/Lys membrane contributes to the nutrient input that promotes oncogenic mTORC1 signalling to drive anabolic pathways for cancer growth.

In the nearness of NPC1/2, lysosomal-associated membrane proteins 1/2 (LAMP 1/2) represent major LE/Lys membrane constituents, capable of binding cholesterol, participating in cholesterol transfer between NPC2 and NPC1 and, consequently, LE/Lys-Chol egress [12,92,93,94]. LAMP1/2 are critical for lysosomal integrity, and have multiple pro-invasive and metastatic, but also tumor suppressor roles, which have been reviewed in detail [95,96]. Several LAMP-related molecular events relevant for cancer, such as exocytosis, matrix metalloprotease (MMP) secretion, chaperone-mediated autophagy or mTORC1 signalling [12,94,97], have links to cholesterol homeostasis. Furthermore, LAMP2 deficiency interfered with Rab7 activity, which is central to LE/Lys function [98,99,100] and cholesterol efflux from LE/Lys [12,94] (see also Section 4.3). Elevated LAMP2 levels were found in colorectal, prostate, breast and many other tumors and may elevate autophagy to promote survival pathways and tumor growth [101,102,103,104,105]. Elevated LAMP2 levels in neuroblastoma upregulated autophagy and caused apoptosis, reflecting the diverse outcomes of increased autophagy in cancer settings [106]. In temozolomide-resistant lung cancer cells, LAMP2 expression was downregulated by a microRNA often overexpressed in cancers, indicating a protective role for LAMP2 in drug resistance of lung cancers [107]. However, whether the impact of LAMP1/2 up- or downregulation on cancer cell actions is influenced by the flux of cholesterol through the LE/Lys compartment remains to be clarified.

Recently, another abundant lysosomal integral membrane protein 2 (LIMP2) was identified to bind and facilitate LDL-derived cholesterol transfer to the ER for esterification and storage in lipid droplets [12,108]. Although LE/Lys-Chol egress mediated by LIMP2 was noticeably slower compared to NPC1, it occurred in an NPC1-independent manner [12,108]. Future studies will need to unravel how cells coordinate the cholesterol transporter activities of NPC1, LAMP2 and LIMP2 when LDL-cholesterol supply is high and whether the cholesterol-binding activities of LAMPs and LIMP2 are relevant contributors to the pathophysiology of certain cancers.

### 3.3. NPC1 and Cholesterol Transport Routes to Focal Adhesions

Substantial evidence in the literature points to the majority of LE/Lys-Chol being delivered to the cell surface [109,110]. At the plasma membrane, LDL-derived cholesterol from LE/Lys feeds into the pool of cholesterol that is considered ‘accessible’, based on its ability to interact with cholesterol-binding toxins [109]. This includes cholesterol pools in focal adhesions, which at the leading edge, continuously undergo assembly and disassembly to promote cell migration. For cells to move forward, coordinated delivery of integrins, cell adhesion receptors composed of α and β subunits that bind to extracellular matrix (ECM), and recruitment of signalling proteins such as Src and focal adhesion kinase (FAK) to focal adhesions is essential [111]. Increased integrin cell surface expression, ECM and MMP secretion, and elevated Src and FAK activity significantly contribute to tumor progression [111,112].

Earlier studies based on unphysiological and vigorous cholesterol depletion that disrupt the functional integrity of cholesterol-rich microdomains at the cell surface, supported cholesterol being essential for focal adhesion assembly, recruitment and signalling of integrins, Src, FAK, Ras GTPases and growth factor receptors [113,114,115,116,117]. More relevant for physiological settings, LDL-cholesterol-containing vesicles emanating from LE/Lys were delivered in the vicinity of focal adhesions at the cell surface [69,70]. In these and other studies, LDL stimulated focal adhesion numbers, dynamics and migration, also in cell models with aggressive behavior known to display enhanced integrin recycling, including A431 squamous cell carcinoma [26,69,70,118].

In these A431 cells, LDL-cholesterol transport from LE/Lys to focal adhesions required the recruitment of the GTPase Rab8a to LDL-cholesterol-containing vesicles in an NPC1-dependent manner, followed by the docking of these vesicles to cortical actin close to the cell surface through Rab8a and myosin5b interactions [70]. Furthermore, a member of the ORP protein family, ORP2, by promoting the bidirectional exchange of LDL-derived cholesterol and phosphatidylinositol-4,5-biphosphate between LE and recycling endosomes, controlled FAK activation in integrin-containing recycling endosomes, stimulating focal adhesion dynamics and migration in A431 cells [119].

Despite the critical role for NPC1 in delivering LDL-derived cholesterol to focal adhesions in the A431 model for cancer cell motility [70,119], cell-specific differences may exist, as large focal adhesion complexes containing active FAK were observed in NPC1 mutant Chinese hamster ovary (CHO) fibroblast cell lines [69]. These findings indicated an ability to overcome NPC1 deficiency and LE/Lys-Chol deprivation and still enable focal adhesion assembly. It has yet to be determined if de novo cholesterol synthesis or other lipids can replace LE/Lys-Chol in focal adhesions of NPC1 mutant CHO cells [69]. However, cellular distribution of focal adhesions in these NPC1-deficient fibroblasts was altered, which might reflect slower focal adhesion turnover leading to reduced migratory potential upon LDL exposure. Strikingly, restoration of LE/Lys-Chol egress via NPC1-independent transport routes in NPC1-deficient CHO cells was associated with increased cholesterol staining in focal adhesions and reinstatement of LDL-inducible cell migration [69] (see Section 4.4 below).

Other examples for trafficking events sensitive to LE/Lys-Chol and relevant for cancer cell migration, invasion and transformation may cover the translocation of Src tyrosine kinase from LE to focal adhesions, which is critical for the disassembly and turnover of cell–ECM interactions and regulated by the endosomal sorting complexes required for transport (ESCRT) and several SNARE proteins [120,121]. SNAREs are critical regulators of membrane transport, with SNAREs in vesicle membranes (v-SNAREs) binding to SNAREs in target membranes (t-SNAREs) to allow tethering, docking and fusion of membranes and delivery of cargo to its correct destination. Out of the SNARE protein family, syntaxin 7 (Stx7), Stx8, and vesicle-associated membrane protein 7 (VAMP7) and VAMP8 contributed to regulate Src trafficking from LE to focal adhesions. Along the same route, α5β1 integrin recycling drives invasiveness in pancreatic and ovarian cancers [122]. VAMP7 and ESCRT also deliver membrane type 1 metalloproteinase (MT1-MMP) to the cell surface for ECM proteolysis, which is crucial for invasive migration [123]. Thus, several SNAREs regulate cell surface delivery of ECM and integrins, which is fundamental for cell migration and cancer cell metastasis and as outlined below, modulated by cholesterol.

### 3.4. NPC1 Influences SNARE-Dependent Cell Surface Delivery of ECM Proteins and Integrins

Besides the rather direct route from LE/Lys to focal adhesions via recycling endosomes (Section 3.3) [70,119], earlier studies have implicated NPC1 to feed into the trafficking of LE/Lys-Chol via the ER to the trans-Golgi network (TGN), requiring the SNAREs Stx6, Stx16 and VAMP4 [124]. Once in the TGN, cholesterol could then be delivered to the plasma membrane by secretory pathways and/or the communication with recycling endosomes [71,125,126,127].

These cholesterol transport routes could indirectly influence cancer cell growth and motility. For instance, NPC1 inhibition interfered with cholesterol-sensitive vesiculation events in the Golgi apparatus, inhibiting caveolin-1 (cav-1) transport from the Golgi to the cell surface and, consequently, caveolae formation [126,127]. Cav-1 has well-established tumor suppressor and promoter activities in many cancers with links to drug resistance [128,129,130]. Mechanistically, NPC1 inhibition caused cholesterol depletion in the Golgi apparatus, which interfered with the recruitment of cytoplasmic phospholipase A2 and its cholesterol-dependent ability to release cav-1-containing secretory vesicles from the TGN [126,127]. As cholesterol drives cav-1 export from the Golgi [131], NPC1-dependent delivery of LDL-derived cholesterol to the Golgi could modulate cav-1-trafficking kinetics to form caveolae, and their role to serve as signalling platforms that transmit oncogenic signals [128,129,130]. In support of this, NPC1 mutant-like cancer cell models revealed changes in the plasma membrane order, reinforcing direct or indirect cell surface delivery of LE/Lys-Chol to provide structural integrity of membrane microdomains and signalling complexes [132].

In addition, several cholesterol-sensitive SNAREs [10,71,133,134,135,136] that regulate exocytic vesicular transport routes to the plasma membrane were affected by the loss of supply with LE/Lys-Chol due to NPC1 inhibition. The majority of cellular cholesterol (70–80%) is found at the plasma membrane and unphysiological cholesterol depletion using methyl-β-cyclodextrin disintegrated plasma membrane clusters enriched in the t-SNAREs Stx4 and soluble N-ethylmaleimide-sensitive fusion protein 23 (SNAP23). Likewise, pharmacological or genetic NPC1 inhibition depleted cholesterol at the plasma membrane, which was accompanied by the mislocalization and dysfunction of Stx4 and SNAP23 [136]. Upon blockage of LE/Lys-Chol egress, both SNAREs were not associated with raft-like structures at the plasma membrane, but accumulated in the Golgi apparatus [136]. Most relevant for cancer cell motility, this correlated with a strongly reduced secretion of cargo along the exocytic pathway, including fibronectin (FN), an ECM protein [136].

Thus, LE/Lys provide the source for cholesterol pools at the plasma membrane that contain and stabilize SNAP23/Stx4-containing membrane domains. This is likely to strengthen the adhesive and migratory properties of cancer cells, where enrichment of ECM proteins such as FN supports cancer cell adhesion, directional migration towards FN [137] and signalling cascades that promote migratory activities in oncogenic settings [111,112]. Expression of SNAP23, Stx4 and other SNARES are often upregulated during the progression of cancers [138], indicating that LDL-cholesterol supports invasive potential via multiple Stx4/SNAP23-dependent trafficking events that are often de-regulated in cancer metastasis. These include cell surface presentation of integrins and MMPs, and activation of tyrosine kinases for oncogenic signalling at focal adhesions [137,139,140,141].

LE/Lys-Chol also affects migratory and invasive behavior regulated by SNARE-dependent recycling of integrins, which bind to ECM to enable forward cell movement. It has long been known that cholesterol levels at the plasma membrane influence adhesion, migration and integrin-dependent activation of signalling cascades [113,114,115,116,117]. In fact, the ability of SNAREs to bind cholesterol [135] may support SNARE-mediated internalization and recycling of integrins at the leading edge for cells to move forward [111,112]. Pointing to LE/Lys-Chol to assist in this process, pharmacological or genetic NPC1 inhibition reduced cholesterol levels in recycling endosomes and concomitantly, strongly diminished integrin transport back to the cell surface. Using several cancer cell models, mislocalization of the SNARE protein Stx6 was identified as an underlying cause. Stx6 is normally located in recycling endosomes, but accumulates in the Golgi apparatus upon NPC1 inhibition, hindering its role to facilitate the delivery of integrins to the cell surface [68,71,134]. Consequently, LDL-cholesterol inducible cancer cell migration and invasion in 2- and 3-dimensional environments were compromised [68,69,71,134]. These Stx6- and LE/Lys-Chol-dependent events relevant for cancer cell motility might require cooperation with Rab11, which also stimulates β1 integrin recycling [142], and influences cholesterol homeostasis in recycling endosomes [143].

Thus, elevated Stx6 levels in breast, liver and prostate cancers [139] might reflect the need for cancer cells to establish fast and efficient transport that accommodates and couples higher cholesterol fluxes with integrin recycling kinetics. This might extend beyond Stx6-dependent integrin recycling, as Stx6 also participates in LE/Lys-Chol transport to the ER via the TGN and in secretory pathways emanating from the Golgi [124,144].

## 4. Potential Roles of LE/Lys-Chol Transfer across Membrane Contact Sites for Cancer Cell Actions

The majority of LE/Lys-Chol is delivered to the cell surface (Figure 1), and from there is transported back to the ER to regulate the feedback mechanisms that govern cholesterol homeostasis [109,110]. In addition, direct transport of LDL-derived cholesterol from LE/Lys to the ER has been described [10,11], with both routes probably supplying cholesterol to the Golgi and affecting cholesterol-sensitive cell surface delivery of cav-1, ECM proteins and integrins (see Section 3). In addition, once in the ER, LDL-derived cholesterol can be esterified by ACAT1 for storage as cholesteryl ester in lipid droplets, serving as an important source of cholesterol in cancer cell growth, as shown for prostate and pancreatic cancers [13,60,61].

Most relevant for LDL-cholesterol transport from LE/Lys to the ER, the discovery of cholesterol transfer across membrane contact sites (MCS) has created great momentum to address how organelles communicate to transfer cholesterol. MCS are specialized small areas of close apposition between two organelles and at the LE/Lys-ER interface, lipid and cholesterol transfer proteins, together with tethers, sorting nexins, membrane channels, SNAREs, small Rab-GTPases and annexins mediate ion and lipid exchange [9,10,11,12,145,146]. It would go beyond the scope of this review to list all MCS-associated proteins, and we recommend excellent reviews that address the association of MCS with cancer-related events for further reading [147,148,149,150]. Most important here, NPC1-dependent and -independent cholesterol transfer mechanisms across MCS between LE/Lys and the ER involve members of the StARD and ORP families, Rab7 and their regulators and effectors, as well as the scaffolding protein annexin AnxA6 [9,10,11,12,145,146], all of which with links to cancer that will be described in more detail in the following sections.

### 4.1. StARD3

StARD3 (or metastatic lymph node 64, MLN64) is found in LE, and, like other members of the StARD family, can bind and transport cholesterol between organelles. StARD3 has been identified at the LE/Lys-ER interface and may act as a tether for the formation of MCS for cholesterol transfer [151]. However, the direction of StARD3-mediated cholesterol transfer across MCS between these two organelles is not fully understood and may depend on nutritional status and cholesterol availability. Some studies implicated StARD3 to move cholesterol from the ER or plasma membrane into LE [151,152,153,154,155,156]. On the other hand, in NPC1 mutant CHO cells, StARD3 contributed to the restoration of LE/Lys-Chol egress via Rab7-dependent cholesterol transfer to the ER [9,157] (see below). However, in NPC patient fibroblasts, StARD3 overexpression did not rescue LE/Lys-Chol accumulation [152,153] and in macrophages or fibroblasts, did not increase cholesterol esterification in the ER [154,158]. In addition, StARD3 has been linked to cholesterol overload in mitochondria in NPC1 deficiency [159,160], which supported roles for StARD3 in LE/Lys-Chol transport to mitochondria for steroidogenesis, mitochondrial well-being and energy production, all anti-apoptotic properties in cancer settings [161,162].

In humans, the StARD3 gene is localized on chromosome 17, within 50 kilobases of the epidermal growth factor receptor 2 (HER2, ERBB2) [163,164]. Hence, StARD3 is often (~25%) co-amplified with HER2 and highly expressed in breast carcinomas [163,164,165,166,167,168]. Elevated StARD3 levels correlated with poor overall survival, disease metastasis-free survival and relapse-free survival in HER2-positive breast cancer [163,166,169] and a lower response to trastuzumab therapy [166,170,171]. In contrast, low StARD3 levels predicted poor prognosis and overall survival in ER-positive and triple-negative breast cancers (TNBC) [166] (Table 3). Although the direction of cholesterol transfer mediated by StARD3 and its potential contribution to intratumoral steroid production in estrogen-responsive tumors is still unclear, elevated StARD3 expression may promote breast cancer cell migration and invasion [169,172,173,174]. In contrast, StARD3 depletion decreased HER2-positive breast cancer cell proliferation and cell cycle progression [175,176]. Of note, StARD3-dependent cholesterol transfer in these cancer-related findings was not addressed. However, the StARD3-dependent restoration of LE/Lys-Chol export in NPC1 mutant cells lacks the gatekeeper AnxA6, leading to increased amounts of cholesterol in focal adhesions and cholesteryl ester storage, correlated with improved LDL-inducible migratory activities (see Section 4.4 below) [69,157].

Elevated StARD3 levels were also found in ovarian cancer [179], associated with increased risk of pancreatic cancer [183] and short relapse-free time in prostate cancer [180]. Like breast cancer, StARD3 was co-amplified with HER2 in gastric cancer [177,178] and correlated with poor prognosis [177]. Interestingly, the fusion of StARD3 with the gene encoding protein phosphatase 1 regulatory inhibitor subunit 1B in >20% of primary human gastric cancers might increase cell proliferation through activation of the PI3K/Akt pathway [184]. In gastric cancer, StARD3 upregulation was proposed to stimulate cholesterol transfer to mitochondria to activate steroidogenesis [178] and improve mitochondrial homeostasis, accelerating cancer cell survival [185].

Recently, the in silico development of the StARD3 inhibitor VS1, which has moderate potency but specifically induced StARD3 degradation, reduced cell viability of breast and colon cancer cell lines [182]. Other therapeutic approaches included StARD3 depletion in combination with lapatinib, which prominently reduced cell viability and proliferation, and increased apoptosis in breast cancer cells [176] (Table 3).

### 4.2. ORP Proteins

The large ORP protein family of lipid-binding/transfer proteins comprises several members that have the capacity to transfer two lipids in opposite directions over MCS that connect LE/Lys with other organelles [186].

OSBP is the founding member of the ORP family and can deliver cholesterol from the ER to the limiting LE/Lys membrane in exchange for phosphatidylinositol-4-phosphate moving in the opposite direction. This transport route may contribute to mTORC1 hyperactivation in NPC1-deficient cells as pharmacological inhibition or depletion of OSBP downregulated mTORC1 signalling, and restored defective autophagy in NPC mutant cells [91].

ORP1L interacts with the small GTPase Rab7, its effector Rab7-interacting lysosomal protein (RILP) in LE and with VAMP-associated proteins in the ER. While these interactions are known to determine sterol-dependent motility of LE vesicles [187,188,189], MCS numbers between LE/Lys and the ER [190,191,192] and final stages of autophagy [193], the function of ORP1L in cholesterol transport is not fully understood. In the common HeLa cervical cancer cell line, low LDL-cholesterol levels in endosomes were a prerequisite for ORP1L to facilitate cholesterol transport from the ER to LE [194,195]. This supported organelle tethering via the annexin A1 (AnxA1) protein in order to ensure the proper targeting of epidermal growth factor receptor (EGFR) for lysosomal degradation. However, in HeLa and human embryonic kidney cells (HEK293) ORP1L may also deliver cholesterol in the opposite direction (LE/Lys to ER) as ORP1L depletion caused LE/Lys-Chol accumulation, reduced cholesterol esterification, and increased de novo cholesterol synthesis [196]. In line with these findings, ORP1L overexpression reduced LE/Lys-Chol accumulation in NPC1 mutant CHO cells [160]. However, the latter was also observed upon ectopic overexpression of an ORP1L deletion mutant lacking its sterol-binding domain, which may indicate that other functions, such as MCS formation, rather than the cholesterol-binding capacities of ORP1L, may rescue the NPC1 mutant phenotype [160]. In support of this, in NPC1 mutant fibroblasts, the adenoviral RIDα protein can take advantage of either ORP1L or the ORP1L mutant defective in sterol-binding to restore LDL-cholesterol egress to the ER, followed by cholesterol esterification for storage in lipid droplets [197]. Taken together, one can speculate that physiological conditions and cell-specific repertoires of cholesterol transporters determine the direction of ORP1L-mediated cholesterol transfer between LE/Lys and the ER.

Besides OSBP and ORP1L, ORP2 can exchange LDL-derived cholesterol with phosphatidylinositol-4,5-biphosphate between LE and recycling endosomes. This assisted FAK activation in integrin-containing recycling endosomes, stimulated focal adhesion dynamics and migration in A431 squamous carcinoma [119] and might contribute to explaining the inhibitory effects of ORP2 depletion on proliferation, migration, invasion and Akt signalling in hepatocytes [198,199]. In addition, ORP5 in the ER was reported to interact with NPC1 and remove cholesterol from the limiting membrane of LE/Lys [200]. However, ORP5 also contributes to lipid transfer between other organelles and its role in phosphatidylserine delivery to the plasma membrane may indirectly influence LE/Lys-Chol egress [110,186].

Hence, several ORPs participate in cholesterol egress from LE/Lys that connects to many cellular activities relevant for tumor initiation, growth and progression. The ability of the abovementioned ORPs to modify the flux of cholesterol and further lipids between LE/Lys and other organelles could impact signalling cascades driven by mTORC1, EGFR, phosphoinosites and FAK, alter integrin cell surface delivery and focal adhesion dynamics, and determine autophagic activities. Up to date, ORP3, ORP4 and ORP5 have been described to support tumor growth and metastatic properties [186,201,202,203], and future studies will need to clarify whether ORPs in LE/Lys contribute to cancer growth and progression. Moreover, the anticancer efficacy of the antifungal itraconazole, which also inhibits NPC1 and is being repurposed in clinical trials for several cancers (see Section 3.1 and Table 2), could be potentiated by its ability to bind and inhibit the OSBP- and ORP4-mediated exchange of cholesterol and phosphatidylinositol-4-phosphate. This feature provides itraconazole with a broad inhibitor spectrum against enteroviruses [204] and together with several other OSBP inhibitors with antiviral properties, one can speculate this may also have potential in LE/Lys-Chol -driven tumor growth and progression [205].

### 4.3. Rab Proteins

Rab proteins belong to the Ras superfamily of small GTPases and undergo cycles of GTP/GDP exchange, which is regulated by guanine-nucleotide exchange factors and GTPase-activating proteins [206]. While inactive Rabs (GDP-bound) remain cytosolic, membrane-anchoring (prenylation) enables stable membrane association of active Rabs (GTP-bound) in a specific location. Each Rab protein interacts with a variety of downstream effectors to regulate the directional movement of vesicles and ensure proper and organelle-specific functioning, with Rab7 being the master regulator of the LE/Lys compartment [206]. Within endosomes, the activities of several Rabs are sensitive to changes in cholesterol levels within their membrane microenvironment. For example, elevated LE/Lys-Chol in NPC mutant cells caused sequestration of inactive Rab9 on LE/Lys membranes, disrupting membrane transport between LE and the TGN [207]. Similarly, excess cholesterol in early endosomes interfered with the Rab4-dependent recycling of ligands back to the cell surface [125]. Likely in concert with LE/Lys-Chol transporters, several Rab GTPases coordinate LE/Lys-Chol transport to cellular sites with critical roles in cancer cell growth and motility via vesicular trafficking or cholesterol transfer across MCS.

For instance, Rab8 overexpression rescued LE/Lys-Chol accumulation in NPC1-deficient cells [208]. Follow-up studies then identified Rab8a to interact with myosin5b to facilitate the docking of LDL-cholesterol-containing vesicles emanating from LE/Lys in an NPC1-dependent manner to the cell surface, stimulating focal adhesion dynamics and migration in A431 carcinoma cells [70,119]. Rab11 was also implicated in LE/Lys-Chol transport along recycling pathways and controlling β1 integrin recycling [142,143], but it did not influence Rab8-regulated delivery of LDL-derived cholesterol to the recycling of endosomes and focal adhesion turnover [70]. In addition, Rab9 could overcome NPC deficiency and contribute to LE/Lys-Chol transport to the TGN [125,207,209].

Thus, one can envisage that elevated expression and activity of these Rab proteins could raise the kinetics of LE/Lys-Chol fluxes that feed into a mechanism to increase biomass and aggressiveness. Vice versa, increased cholesterol delivery to Rab-containing microdomains could affect their function and GTPase activity [125,207,208,209,210]. This reciprocal relationship between Rab proteins and cholesterol could be relevant for many cancer-related aspects. In fact, Rab GTPases found in endosomal compartments are differentially expressed in many cancers, with multiple roles in cancer cell motility and MMP secretion [211,212,213].

In addition to the Rab proteins mentioned above, Rab7 appears most critical for LE/Lys-Chol homeostasis. At the LE/Lys limiting membrane, active Rab7-GTP recruits effector proteins that ensure the integrity and proper functioning of the LE/Lys compartment [9,11,206,210,214]. This includes the delivery of LDL along the endocytic pathway to LE/Lys [215] and subsequent distribution of LDL-derived cholesterol from LE/Lys to other organelles [9,11]. Earlier studies revealed LE/Lys-Chol accumulation upon NPC1 inhibition to increase Rab7 amounts in LE/Lys, interfering with Rab7 activity and Rab7-dependent LE/Lys motility [210]. In line with these observations, Rab7 depletion compromised LE morphology, and affected NPC1-dependent and -independent export routes of LDL-derived cholesterol from LE/Lys to other cellular sites [9,157,214]. Vice versa, upregulation of Rab7-GTP levels not only rescued cholesterol accumulation in NPC1 mutant cells [9,157,214,216], but was also associated with LDL-inducible migration and invasion of cancer cells [69]. One can envisage that underlying mechanisms may include Rab7 to support vesicular transport of LDL-containing LE vesicles to Rab8-regulated integrin- and FAK-containing recycling endosomes, with ‘kiss and run’ contacts between these organelles enabling sterol exchange [70,119]. Alternatively, Rab7-dependent cholesterol transfer across MCS to the ER involving either NPC1, StARD3 or ORP1L, could feed into cholesterol pools in lipid droplets and the plasma membrane that support oncogenic behavior [9,11,157,187,188,190].

Indeed, Rab7-related activities influence cell growth and motility and both tumorigenic and anti-tumorigenic effects have been reported [217,218]. For example, upregulated Rab7 levels were documented in ovarian, thyroid and peritoneal serous carcinoma [219,220], and were responsible for endothelial tumor growth and metastasis caused by metabolic reprogramming in a mouse model for lysosomal lipase deficiency [221]. Rab7 has also been associated with lipid metabolic signalling, mTORC1 activity, Rac1 GTPase-dependent lung cancer cell migration, and anti-apoptotic Akt signalling in breast cancer [222,223,224,225]. In support of elevated Rab7 levels conferring tumor promoter activities, overexpression of a dominant-negative Rab7 mutant inhibited migration and invasion of cervical and fibrosarcoma cell lines [140]. In contrast, loss of Rab7 function promoted invasive actions in prostate cancer and glioblastoma [226,227], contributed to oncogenic EGFR signalling and tumor growth in thyroid cancers [228], elevated angiogenesis and proliferation in A549 lung cancer cells [229]. In relation to anticancer drug efficacy, Rab7 was downregulated in several cisplatin-resistant cervical cancer cell lines and Rab7 overexpression re-sensitized cisplatin-resistant cells [230]. Hence, tumor promoter and suppressor roles for Rab7 in different cancers exist, with varied Rab7 expression levels even along multiple stages during melanoma progression [231] and inflammatory breast cancer [232]. Recently, the antimalarial drug mefloquine hydrochloride was identified to inhibit Rab7 (and Rab5), providing promise to eliminate colorectal cancer stem cells [233]. Furthermore, statin-mediated inhibition of Rab7 prenylation or blocking nucleotide binding using the Rab7 inhibitor CID-1067700 showed antitumor potential in TNBC and epithelial ovarian cancer cell lines [234,235]. Further research will need to determine if these observations are linked to cholesterol homeostasis (Table 4).

### 4.4. Annexin A6

AnxA6 is the largest member of the conserved annexins, a protein family with a modular domain organization that supports interactions with a plethora of proteins and lipids [236,237]. Together with their Ca^2+^-regulated ability to bind membranes, this enables AnxA6 and other annexins to act as scaffolding proteins, supporting signal complex assembly, membrane and cholesterol transport, microdomain formation, and cytoskeleton rearrangements, all relevant for biological processes related to cell proliferation and motility [32,236,237,238,239].

AnxA6 preferentially binds negatively charged phospholipids in a Ca^2+^-dependent manner. This probably includes membrane domains such as clathrin-coated pits, coupling AnxA6 to EGFR and possibly other growth factor receptors, which signal at the cell surface and from endocytic compartments, with oncogenic potential to accelerate cell growth and motility. Indeed, at the plasma membrane, AnxA6 facilitates membrane recruitment of protein kinase Cα (PKCα) and the GTPase activating protein p120GAP, both negative regulators of the EGFR and the Ras/mitogen-activated protein kinase pathway [240,241,242]. In addition, AnxA6 affects cell viability and motility through interaction with several Src family kinases, and other regulatory circuits that control the cell cycle, Ca^2+^ homeostasis, membrane repair, glucose and lipid metabolism, mitochondrial homeostasis and pH sensing. Of note, this also appears to include extracellular AnxA6 activities in TNBC, PDAC and gastric cancer, affecting focal adhesion dynamics, metastatic behavior and response to therapy. Several review articles from our group and others have extensively covered AnxA6-related aspects relevant to cancer growth and metastasis [32,236,237,238,239,243,244].

Hence, overexpression and gene knockdown studies in cell culture and animal models, as well as expression patterns in patient cohorts, identified AnxA6 to exhibit tumor suppressor activities. For instance, AnxA6 overexpression promoted PKCα- and p120GAP-mediated EGFR and Ras inactivation, respectively, inhibiting anchorage-independent growth of EGFR overexpressing and ER-negative breast cancer cell lines, A431 migration, invasion and A431 xenograft growth. Furthermore, elevated AnxA6 scaffold levels contributed to improving the efficacy of tyrosine kinase inhibitors (TKIs) targeting EGFR to reduce growth, migration, and invasive properties of EGFR overexpressing A431 carcinoma cells [240,241,242,245].

This and additional scaffolding functions that inhibit oncogenic signalling cascades could also contribute to AnxA6 tumor suppressor functions in other cancers with diagnostic value for tumor malignancy and progression [32,244,246]. Indeed, early onset and rapid growth of tumors derived from AnxA6-depleted TNBC cells were in line with low AnxA6 levels and poor overall survival of basal-like TNBC patients [239,247]. Overall, AnxA6 was downregulated in highly aggressive TNBC subtypes [239,247], gastric [248] and cervical cancer [249], as well as HCC [250] (Table 5).

On the other hand, tumor promoter activities of AnxA6 have also been reported, with AnxA6 displaying pro-invasive functions in invasive breast cancer cells [239,246]. In gastric cancer, AnxA6 conferred drug resistance via β1 integrin and FAK activation [259]. Elevated AnxA6 levels were also documented during the progression of pancreatic cancer [254,255,256], women’s thyroid cancer [257], squamous cervical cancer [251], ovarian carcinoma [253], esophageal adenocarcinoma [260] and melanoma [252].

In regard to the potential of AnxA6 levels as a marker to predict cancer recurrence and chemotherapy response, its downregulation sensitized TNBC cells to EGFR-TKIs and was associated with poorer overall and distant metastasis-free survival [239,247]. Vice versa, AnxA6 upregulation in TNBC cells modulated the efficacy of cytotoxic and/or EGFR-targeted therapies and the development of drug resistance [246,247,261]. Hence, differential expression patterns of AnxA6 expression levels and its interaction partners will likely result in cell-specific intra- and extracellular scaffolding functions, contributing differently to the progression and treatment outcome of the various cancers [32,238,239,244] (Table 5).

Most relevant for cholesterol homeostasis, AnxA6 expression levels significantly impacted LE/Lys-Chol egress, with consequences for cells to grow or move forward and invade [32,134,157]. In earlier studies, we and others identified AnxA6 to support endocytosis and targeting of LDL to lysosomes [258,262,263,264].

The generation of cholesterol-rich LE/Lys membranes through prolonged LDL loading or genetic/pharmacological NPC1 inhibition triggered the translocation of AnxA6 proteins to LE/Lys [262,265,266], indicating cholesterol-binding properties of AnxA6, which were recently confirmed in binding studies in vitro [135]. Importantly, AnxA6 overexpression resulted in LE/Lys-Chol accumulation that resembled loss-of-NPC1 function, interfered with cytoplasmic phospholipase A2-dependent transport of cav-1 to the cell surface, and caused mislocalization and dysfunction of the SNAREs proteins Stx4, SNAP23 and Stx6 [126,127,134,136]. Consequently, like NPC1 deficiency, elevated AnxA6 levels reduced cholesterol-sensitive caveolae formation, FN secretion and integrin recycling [127,134,136], effectively reducing LDL-inducible migration and the invasion of CHO and A431 carcinoma cells [69,134,245].

Strikingly, AnxA6 depletion in NPC1 mutant cells restored cholesterol efflux from LE/Lys in a Rab7-dependent manner [157], which coincided with increased cell motility and association of the cholesterol biosensor D4H with active FAK at cell edges [69]. Hence, AnxA6-regulated cholesterol transport routes emanating from LE/Lys seem to contribute to cholesterol delivery to focal adhesions, thereby improving migratory activities [69]. The role of AnxA6 as a gatekeeper of LE/Lys-Chol distribution to focal adhesions for cell migration and lipid droplets for storage could reflect aspects of cholesterol-related anticancer drug resistance [3,4,13,14,15,16], as prolonged exposure of TNBC cells to lapatinib or other TKIs targeting EGFR was accompanied by LE/Lys-Chol accumulation, AnxA6 upregulation and the development of drug resistance [239,267].

## 5. Conclusions

In this review, we have provided an overview of the expression profiles and activities of cholesterol transporters, regulatory and scaffolding proteins in LE/Lys that may improve the delivery of LDL-cholesterol to other cellular organelles, fostering cancer growth, progression and in many settings, contributing to the development of drug resistance. Besides the cholesterol transporters listed here, earlier reports have described the localization of the scavenger receptor BI and ATP binding cassette transporters A1 and G1 in LE/Lys, contributing to LE/Lys-Chol egress [268,269,270]. However, as their predominant location and activity appears rather at the plasma membrane, we recommend reviewing articles that cover these transporters in the context of cancer growth, progression and drug resistance for further reading [173,271].

Overall, increased LDL-cholesterol uptake mediated by LDLR upregulation, possibly accompanied by elevated expression and activity of LE/Lys-Chol transporters (NPC1, StARD3, and ORPs), lysosomal LE/Lys-Chol binding proteins (LAMP2, LIMP2) and regulators (Rab GTPases), often coincides with increased cancer growth, metastatic behavior and chemoresistance. Loss of scaffolding proteins such as AnxA6 and other yet unidentified players that normally may act like guardians to limit LE/Lys-Chol transport activities could further promote LE/Lys-Chol driving oncogenic actions. Beyond the co-amplification of StARD3 with HER2 in breast and gastric cancers (Section 4.1), it has yet to be identified if gene mutations or polymorphisms of any of these proteins listed above have functional consequences for LE/Lys-Chol export that may contribute to oncogenic behavior and drug efficacy. A substantial number of publications summarized in this article are based on preclinical observations from cell culture or animal models and will require further validation using data sets from patient cohorts. Nevertheless, experimental approaches to inhibit LE/Lys-Chol transport targeting LDLR, NPC1 or StARD3 and small molecules inhibiting ORP proteins and Rab7 GTPase provide promise for therapeutic advances, and interfering with LDL-cholesterol distribution from LE/Lys could become a therapeutic target for cancer treatment and reduce the risk for metastasis or development of drug resistance.

## Figures and Tables

**Table 1:** Characteristics and roles of LDLR in cancer.

LDLR Upregulation and Tumorigenic Outcomes	Cancer Types
Elevated LDLR expression	breast cancer [26,49,50], colorectal cancer [32,33], glioblastoma [35], HCC [28], lung cancer [30,31], leukemia [36,51], lymphoma [29], nasopharyngeal carcinoma [34], renal cancer [37], PDAC [24,25]
Promoting proliferation, migration and invasion	breast cancer [52], colorectal cancer [38], nasopharyngeal cancer [34], PDAC [25], prostate cancer [53,54,55]
Poor prognosis and clinical outcomes	AML [56], breast cancer [52], cervical cancer [48], HCC [46], PDAC [57,58], Prostate cancer [47]
Chemoresistance	breast cancer [52], ovarian cancer [59], PDAC [25]

**Table 2:** Roles of NPC1 in cancer.

Contribution of NPC1 to Tumorigenic Outcomes	Cancer Types
Upregulation and cancer risk	esophageal cancer [65,74], HCC [75]
Promoting proliferation, migration and invasion	A431 squamous carcinoma [68,69,70,71], cervical cancer [67]
Poor prognosis	ER-negative breast cancer [66], glioma [64]
Chemoresistance	breast cancer [76], leukemia [70,73]
Therapeutic target	Itraconazole: basal cell carcinoma [77], non-small cell lung cancer [78], pancreatic cancer [79], prostate cancer [80] Cepharanthine: head and neck cancer [81], prostate cancer [82] Leelamine: metastatic melanoma [83,84]

**Table 3:** Characteristics and roles of StARD3 in cancer.

Contribution of StARD3 to Tumorigenic Functions	Cancer Types
StARD3 expression and cancer risk	breast cancer [166], gastric cancer [177,178], ovarian cancer [179]
StARD3 expression and poor prognosis	breast cancer [169], ER-positive and triple-negative breast cancer [166], gastric cancer [177], prostate cancer [180]
Predictor for chemotherapy response	breast cancer [169,181]
Therapeutic target	compound VS1: breast and colon cancer [182]

**Table 4:** Roles of Rab7 in cancer.

Contribution of Rab7 to Tumorigenic Outcomes	Cancer Types
Tumor promotor	A431 squamous carcinoma [69,225], lung cancer [224], breast cancer [225], cervical carcinoma [140], ovarian cancer [220], peritoneal serous carcinoma [220], thyroid cancer [219]
Tumor suppressor	A549 lung cancer [229], glioblastoma [227], thyroid cancer [228]
Oncojanus	inflammatory breast cancer [232], melanoma [231]
Cisplatin chemoresistance	cervical cancer [230]
Therapeutic target	mefloquine hydrochloride: colorectal cancer stem cells [233]
	Statins: TNBC, epithelial ovarian cancer cell lines [234,235]. CID-1067700: epithelial ovarian cancer cell lines [235].

**Table 5:** Roles of AnxA6 in cancer.

Contribution of AnxA6 to Tumorigenic Outcomes	Cancer Types
Tumor promotor	breast cancer [246], cervical cancer [251], esophageal cancer [252], melanoma [252], ovarian cancer [253], pancreatic cancer [254,255,256], women’s thyroid cancer [257]
Tumor suppressor	A431 epithelial carcinoma [240,241,242,245], breast cancer (TNBC, EGFR overexpressing and ER-negative) [239,240,241,246], cervical cancer [249], gastric cancer [248], HCC [250]
Chemotherapy response	TNBC [239,247,258], gastric cancer [259]

## Data Availability

Not applicable.

## Abbreviations

ACAT1: acetyl-CoA cholesteryl acyltransferase 1; AML, acute myeloid leukemia; Akt, protein kinase B; AnxA6, annexin A6; EGFR, epidermal growth factor receptor; ER, endoplasmic reticulum; FN, fibronectin; HCC, hepatocellular carcinoma; HER2, human epidermal growth factor receptor 2; LAMP2, lysosome-associated membrane protein 2; LDL, low-density lipoprotein; LDLR, LDL receptor; LE, late endosomes; LIMP2, lysosomal integral membrane protein 2; Lys, lysosomes; MCS, membrane contact sites; mTORC1, mammalian target of rapamycin complex 1; NPC1/2, Niemann–Pick Type C1/2; OSBP, oxysterol-binding protein; ORP, OSBP-related protein; ORP1L, OSBP-related protein 1L; PDAC, pancreatic ductal adenocarcinoma; PI3K, phosphatidylinositol-3-kinase; SNARE, soluble N-ethylmaleimide sensitive factor attachment protein receptor; StARD3; steroidogenic acute regulatory protein-related lipid transfer domain containing 3; Stx, syntaxin 6; TGN, trans-Golgi network; TKI, tyrosine kinase inhibitor; TNBC, triple-negative breast cancer.
